# Advance directives: cancer patients' preferences and family-based decision making

**DOI:** 10.18632/oncotarget.17525

**Published:** 2017-04-28

**Authors:** Yan-Fang Xing, Jin-Xiang Lin, Xing Li, Qu Lin, Xiao-Kun Ma, Jie Chen, Dong-Hao Wu, Li Wei, Liang-Hong Yin, Xiang-Yuan Wu

**Affiliations:** ^1^ Department of Nephrology, First Affiliated Hospital of Jinan University, Guangzhou 510630, People's Republic of China; ^2^ School of Medicine, Jinan University, Guangzhou 510632, People's Republic of China; ^3^ Department of Nephrology, Third Affiliated Hospital of Guangzhou Medical University, Guangzhou 510150, People's Republic of China; ^4^ Department of Medical Oncology, Guangdong Key Laboratory of Liver Disease Research, Third Affiliated Hospital of Sun Yat-sen University, Guangzhou 510630, People's Republic of China

**Keywords:** advance directive, medical decision making, cancer, patients’ preference

## Abstract

**Background:**

Advance directives are a sensitive issue among traditional Chinese people, who usually refrain from mentioning this topic until it is imperative. Medical decisions for cancer patients are made by their families, and these decisions might violate patients’ personal will.

**Objectives:**

This study aimed to examine the acceptance of advance directives among Chinese cancer patients and their families and patient participation in this procedure and, finally, to analyze the moral risk involved.

**Results:**

While 246 patients and their family members refused official discussion of an advance directive, the remaining 166 patients and their families accepted the concept of an advance directive and signed a document agreeing to give up invasive treatment when the anti-cancer treatment was terminated. Of these, only 24 patients participated in the decision making. For 101 patients, anti-cancer therapy was ended prematurely with as many as 37 patients not told about their potential loss of health interests.

**Materials and Methods:**

Participants were 412 adult cancer patients from 9 leading hospitals across China. An advance directive was introduced to the main decision makers for each patient; if they wished to sign it, the advance directive would be systematically discussed. A questionnaire was given to the oncologists in charge of each patient to evaluate the interaction between families and patients, patients’ awareness of their disease, and participation in an advance directive.

**Conclusions:**

Advance directives were not widely accepted among Chinese cancer patients unless anti-cancer therapy was terminated. Most cancer patients were excluded from the discussion of an advance directive.

## INTRODUCTION

An advance directive (AD) is a common procedure for patients with terminal cancer to ensure they die with dignity [1]. In the best of circumstances, the patient, family, and healthcare providers have discussed the treatment options and made the final decision [2, 3]. The concept of an AD is common in Australia, Canada, and the United Kingdom. However, in Confucian areas, there is no system or law to support the decision making of patients [3, 4]. An end-of-life decision is a sensitive issue in China; people, including family members and patients, usually refrain from mentioning this topic until it is imperative [5]. Thus, it is valuable to investigate the practice of ADs in the Chinese population as it might make it easier for Chinese patients in Western countries to make such decisions.

Medical decision making in Western countries is largely individualized. However, this decision-making mode is not prevalent in Confucian areas and the Arab world, including China [6, 7]. In mainland China, medical decision making for patients with malignant disease largely relies on the patients’ families. This mode has always been criticized for its latent moral risks in that the interests of vulnerable patients might not be fully met or, even worse, might be violated by their family members [6, 8, 9]. However, few studies have systematically illustrated the risks of AD decision making in Confucian areas.

Thus, in this multicenter study, we aimed to systematically examine the acceptance of ADs in Chinese cancer patients and their families, to examine patients’ participation in this procedure, and, finally, to analyze the moral risk in the AD decision making for cancer patients in China.

## RESULTS

### Most patients and their family members rejected systematic discussion of AD during anti-cancer therapy

A total of 246 patients and their family members rejected systematic discussion of AD; the majority (95.5%) of these were under anti-cancer treatment. An additional 166 patients or their families accepted the concept of an AD and signed an AD to give up invasive treatment. This decision was made shortly after termination of anti-cancer therapy. Correlational analysis revealed that patients living in villages and who were subordinate members in their families tended to accept ADs. Financial status, gender, age, patients’ educational background, and decision-making style did not influence the acceptance of an AD (Table 1).

**Table 1:** The characteristic of patients accepting AD or not

Characteristics	Non AD group *n* = 246	AD group *n* = 166	*P*
Age	55.3 ± 12.965	57.1 ± 12.911	0.189
Sex			0.582
Female	106 (43.1%)	67 (40.4%)	
Male	140 (56.9%)	99 (59.6%)	
Insurance			0.071
Local	97 (39.4%)	51 (30.7%)	
Non-local	149 (60.6%)	115 (69.4%)	
Main living environment			0.013
Village	100 (40.7%)	88 (53.0%)	
City	146 (59.3%)	78 (47.0%)	
Patient education level			0.089
Ordinary	178 (72.4%)	107 (64.5%)	
Illiterate	68 (27.6%)	59 (35.5%)	
Family status of patients			0.018
Superordinate	91 (37.0%)	43 (25.9%)	
Subordinate	155 (63.0%)	123 (74.1%)	
Decision making mode			0.827
Patient superiority	39 (15.9%)	25 (15.1%)	
Family superiority	207 (84.1%)	141 (84.9%)	
Termination of anticancer therapy	11 (4.5%)	166 (100.0%)	0.000

Abbreviations: AD, advance directive.

### Most cancer patients were excluded from the AD decision making

Of the 166 patients who finally signed an AD, only 24 patients participated in the decision making and signed it according to their own will; the remaining 142 patients were excluded from this decision-making process. Correlational analysis revealed that patients with a better financial situation, living in cities, and with superordinate status in their families tended to participate in the AD discussion, as did those who usually made medical decisions themselves. Nearly all the patients deciding on their own AD knew their entire situation, including diagnosis and prognosis (Table 2).

**Table 2:** The characteristic of cancer patients’ participation in AD decision making or not

Characteristics	Participation *n* = 24	No-participation *n* = 142	*P*
Age	59.2 (24–72)	58.3 (21–85)	0.164
Sex			0.593
Female	8 (33.3%)	59 (41.5%)	
Male	16 (66.7%)	83 (58.5%)	
Insurance			0.048
Local	12 (50.0%)	39 (27.5%)	
Non-local	12 (50.0%)	103 (72.5%)	
Main living environment			0.037
Village	8 (33.3%)	80 (56.3%)	
City	16 (66.7%)	62 (43.7%)	
Patient education level			0.243
Ordinary	18 (75.0%)	89 (62.7%)	
Illiterate	6 (25.0%)	53 (37.3%)	
Family status of patients			0.004
Superordinate	12 (50.0%)	31 (21.8%)	
Subordinate	12 (50.0%)	111 (78.2%)	
Decision making mode			0.000
Patient superiority	13 (54.2%)	12 (8.5%)	
Family superiority	11 (45.8%)	130 (91.5%)	
Patient's awareness of disease			
Diagnosis	24 (100.0%)	94 (66.2%)	0.001
Stage	23 (95.8%)	41 (28.9%)	0.000
Prognosis	23 (95.8%)	32 (22.5%)	0.000
Home hospice	5 (20.8%)	47 (33.1%)	0.231
Recommendation*	8 (33.3%)	57 (40.1%)	0.527

* Termination of anti-cancer therapy was recommended by oncologist. Abbreviations: AD, advance directive.

### Premature ending of anti-cancer therapy induced acceptance of AD

Of the 166 patients who finally signed an AD, 101 terminated anti-cancer therapy prematurely. Correlational analysis revealed that patients with an inferior financial situation tended to terminate anti-cancer therapy prematurely. Similarly, patients with less knowledge of the modern world (i.e., those not living in cities or with lower educational level) tended to end anti-cancer therapy against the recommendation of the oncologist. Moreover, patients receiving full treatment were given more information about their diagnosis. The patients following the doctors’ suggestion intended to die in the hospital. Patients who rejected anti-cancer therapy preferred to die at home (Table 3).

**Table 3:** The characteristic of cancer patients terminating anti-cancer therapy following the recommendation of doctor or not

Characteristics	Recommend *n* = 65	Not recommend *n* = 101	*P*
Age	55.5 ± 14.7	58.0 ± 11.6	0.255
Sex			0.939
Female	26 (40.0%)	41 (40.6%)	
Male	39 (60.0%)	60 (59.4%)	
Insurance			0.024
Local	27 (41.5%)	24 (23.8%)	
Non-local	38 (58.5%)	77 (76.2%)	
Main living environment			0.000
Village	20 (30.8%)	68 (67.3%)	
City	45 (69.2%)	33 (32.7%)	
Patient education level			0.028
Ordinary	49 (75.4%)	58 (57.4%)	
Illiterate	16 (24.6%)	43 (42.6%)	
Family status of patients			0.627
Superordinate	15 (23.1%)	28 (27.7%)	
Subordinate	50 (76.9%)	73 (72.3%)	
Decision making mode			0.752
Patient superiority	11 (16.9%)	14 (13.9%)	
Family superiority	54 (83.1%)	87 (86.1%)	
Patient's awareness of disease			
Diagnosis	53 (81.5%)	65 (64.4%)	0.027
Stage	31 (47.7%)	33 (32.7%)	0.076
Prognosis	23 (35.4%)	32 (31.7%)	0.745
Home hospice	5 (7.7%)	47 (46.5%)	0.000
Decision by patients	8 (12.3%)	16 (15.8%)	0.685

### A great proportion of patients were ignorant of the premature ending of their anti-cancer therapy

Of the 101 patients who prematurely ended anti-cancer therapy, as many as 37 were not told of the rejection of formal medical advice as well as their potential loss of medical interest. None of the 37 patients participated in the AD discussion. Like participation in medical decisions, patients with superior status in the family and those usually making decisions for themselves tended to be given full information about the less optimal medical decision and the consequent prognosis (Table 4).

**Table 4:** The characteristic of cancer patients aware or not of their premature terminating anti-cancer therapy

Characteristics	Not know *n* = 37	Know *n* = 64	*P*
Age	59.5 ± 13.4	57.1 ± 10.4	0.350
Sex			0.210
Female	18 (48.6%)	23 (35.9%)	
Male	19 (51.4%)	41 (64.1%)	
Insurance			0.701
Local	8 (21.6%)	16 (25.0%)	
Non-local	29 (78.4%)	48 (75.0%)	
Main living environment			0.174
Village	28 (75.7%)	40 (62.5%)	
City	9 (24.3%)	24 (37.5%)	
Patient education level			0.175
Ordinary	18 (48.6%)	40 (62.5%)	
Illiterate	19 (51.4%)	24 (37.5%)	
Family status of patients			0.004
Superordinate	4 (10.8%)	24 (37.5%)	
Subordinate	33 (89.2%)	40 (62.5%)	
Decision making mode			0.014
Patient superiority	1 (2.7%)	13 (20.3%)	
Family superiority	36 (97.3%)	51 (79.7%)	
Patient's awareness of disease			
Diagnosis	11 (29.7%)	54 (84.4%)	0.000
Stage	2 (5.4%)	31 (48.4%)	0.000
Prognosis	3 (8.1%)	29 (45.3%)	0.000
Home hospice	14 (37.8%)	33 (51.6%)	0.183
Participation	0 (0.0%)	16 (25.0%)	0.000

## DISCUSSION

Recently, the topic of dignified death and decisions for end-of-life care has received increasing attention worldwide [1, 3]. In decision making involving an AD, family plays a fundamental role in Confucian areas [4]. Family is the functional unit in which members share beliefs and finances based on their culture and experience, develop cohesion between members, and overcome common risks; this has important effects on medical decision making for lethal diseases. In particular, Chinese people tend to depend on their families rather than individuals [6, 8, 10]. Thus, the family plays a much more important role in critical medical decision making than the patient. Another determinant factor in decision making is the financial situation [11, 12]. In mainland China, patients pay a proportion of medical expenses themselves, especially those treated in major cities instead of their hometowns. Consequently, the family paying for the expenses plays the fundamental role in medical decision making instead of the patients. Above all, the family takes a critical role in AD decision making in China. However, the family-based medical decision making style of Confucian areas is disputed for its ignorance of individualism [6, 7, 9, 13]. In this study, we first examined AD decision making in China and then analyzed the latent risks.

Chinese do not like to talk about death, partly due to their superstition that talking about death would cause bad luck and to the shortage of death education in China [12, 14]. The concept of an AD was widely accepted by Chinese oncologists who had received modern education in medicine. However, in most cases, discussion of an AD was not accepted by the family and patient until there was no chance for the patient to receive any anti-cancer therapy or there was a high risk of sudden death of the patient. When an AD had to be discussed, patients living in villages tended to follow the traditional Chinese culture and preferred to die in their family temples. Thus, they more actively accepted the concept of ADs and spent the last days of their lives at home. The superordinate family members tended to make the AD decision for the subordinate members to protect them from fear of death. Summarily, having an AD was widely accepted among Chinese cancer patients and their families; however, it would not be discussed until there was no chance to control cancer.

Most Chinese cancer patients were excluded from the discussion of AD. Only 14.5% participated in the AD decision making. Death education is scarce in traditional Chinese culture and the modern educational system [5, 12, 14, 15], leading to overreacting behavior and mental frustration inpatients with terminal illnesses. As a solution to this difficult situation for patients, Chinese family members always protected the patients from hearing their end-of-life message [5]. Modern Chinese people, mainly living in cities, who have received better education from Western culture tended to participate more in the AD discussion. Moreover, patients with a superordinate family position presented an increased tendency to be involved in their AD discussion. This might be explained by their strong will to fulfill their responsibility to their family members. Despite the above 2tendencies, Chinese families played an important role in making decisions regarding withdrawal of life-sustaining treatment in end-of-life care and preferred to delay providing the information to cancer patients.

Premature ending of anti-cancer therapy induced acceptance of ADs. Traditionally, the paternalistic behavior of physicians has been considered to prevail without question in Asia and the Arab world. The early termination of anti-cancer therapy might be caused by immoral reasons, as suggested in the Methods section. Moreover, the premature ending of anti-cancer therapy might be against the will of the patient. However, he/she may have been told by the family there was no need to continue anti-cancer therapy, which was not the truth. Tensions have been raised between doctors and patients in China [16, 17]. Thus, the oncologist may not be allowed to give information to the patient. If the oncologist disobeys the demands of the family, he/she might be attacked in all forms including complaints to authorities, charges, and verbal and physical attacks of differing intensity. It must be admitted that some premature termination of anti-cancer therapy is done for the good of the patients; for example, the family may have paid too much of what they have to the patient, and the truth of the early ending of anti-cancer therapy may harm the mental happiness of the patients. Above all, the premature ending of anti-cancer therapy was defined as the moral risk of the decision making of ADs for Chinese cancer patients. The spiritual distress the patients experienced when discussing their own ADs was another risk. However, this is difficult to evaluate and was not included in our study.

There was a high incidence (60.8% in the present study) of premature ending of anti-cancer therapy. The major causes of early termination of anti-cancer therapy were financial problems. Additionally, cancer patients living in villages and who had less education considered cancer to be their “fate” [14] and tended to stop fighting against it after failure of the first cycles of anti-cancer therapy. Besides, education level strongly was correlated with the financial situation. Similar to participation in other medical decisions, patients with superior status in the family, who usually made medical decisions themselves, tended to participate more actively in premature termination of anti-cancer therapy. However, most patients were excluded from the determination of premature ending of anti-cancer therapy, and the information was delayed in getting to them.

The present study indicated that the family-based mode of AD decision making might lead to potential moral risk. The premature ending of anti-cancer therapy without telling the patient the truth might be against the health interest of cancer patients and shorten their survival. Thus, the doctors shall improve the education ofthe patients and their family members about their rights and the idealism of hospice care. Besides, the government should give more financial support to cancer patients under palliative treatment.

## MATERIALS AND METHODS

### Patient selection

A total of 412 consecutive adult cancer patients under palliative therapy were included in this study between September 1, 2013, and December 31, 2013, from 9 departments of oncology in local leading general hospitals or cancer centers in 5 big cities across China, including the Third Affiliated Hospital, Cancer Center, and the Sixth Affiliated Hospital of Sun Yat-sen University as well as the Third Affiliated Hospital of Guangzhou Medical University, the Second Affiliated Hospital of GuangDong Pharmaceutical University in Guangzhou, the Fifth Affiliated Hospital of Sun-Yat-sen University in Zhuhai, Dongguan People's Hospital in Dongguan, the First Hospital of Yueyang in Yueyang, and the First Hospital of Shanghai in Shanghai. The following patients were excluded: those whose lives were directly threatened by acute diseases other than cancer and associated complications, those whose cancer diagnosis lacked pathological proof, those who were incompetent in making medical decisions, those with acute leukemia, and those who underwent radical treatment. Additionally, 25 patients were excluded due to a lack of substantial necessary information. This study was approved by the Clinical Ethics Review Board at all the hospitals included in this study. Informed consent was obtained at the time of admission.

### Advance directive procedure

**Figure 1:**
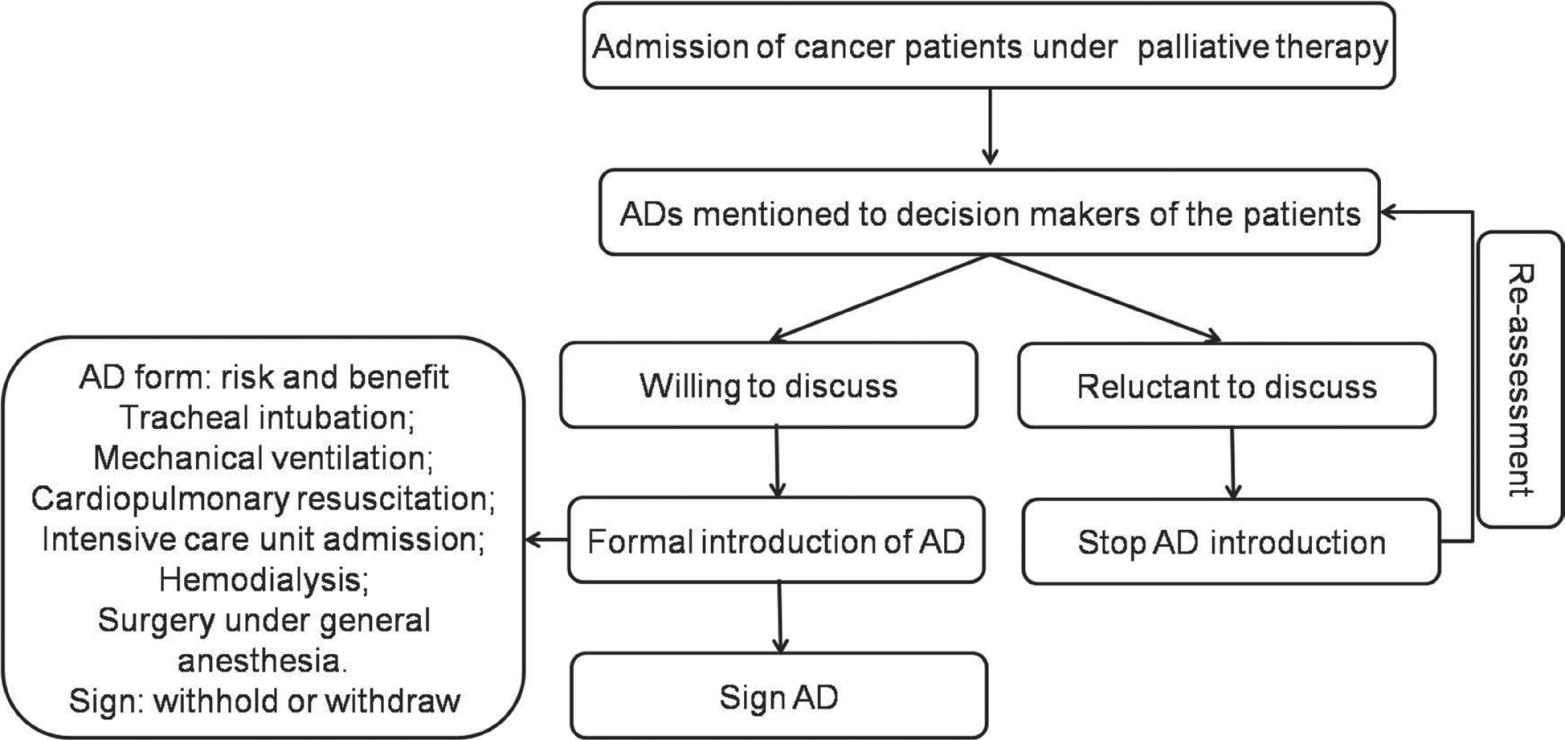
Standard procedure of AD decision making

In most of the hospitals in mainland China, ADs consisted primarily of life supporting therapy and invasive procedures, such as tracheal intubation, mechanical ventilation, cardiopulmonary resuscitation, intensive care unit admission, hemodialysis, and surgery under general anesthesia. Conservative treatments considered AD elements in the Western world, such as transfusion, vasopressor/inotropic, broad-spectrum antibiotics, tube feeding, parenteral fluids, parenteral nutrition, painkiller, simple diagnostic tests, and supplemental oxygen for dyspnea, are not included in ADs in mainland China. An AD was informally mentioned to the main decision makers when the patient was under anti-cancer therapy; if they wished to sign an AD, an AD would be systematically introduced. Routinely, an AD was officially recommended to the main decision makers for each patient (mainly family members and, in limited cases, the patient) when anti-cancer therapy was terminated or there was a high risk of sudden death of the patient (Figure 1).

### Procedure of information collection

**Figure 2:**
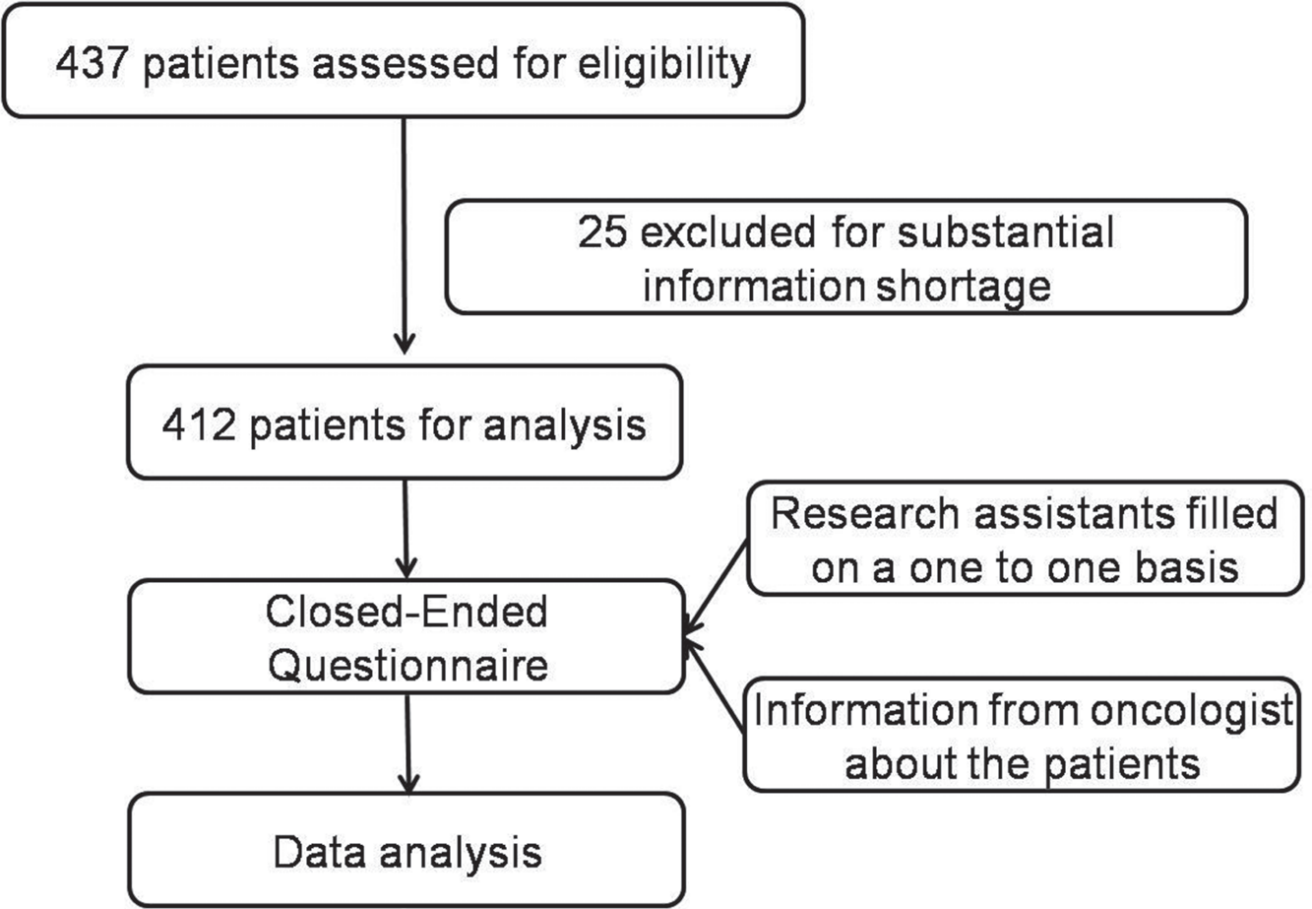
Study design

Three research assistants (oncologists) helped with the data collection. Research assistants were trained to maintain the consistency and guarantee the reliability of the data collection process. The 3 research assistants visited the above hospitals and met with the participant oncologists individually. The information on included patients was provided by their oncologists in charge. Each oncologist was given an explanation of the study purpose and protocol. They were also informed that there was no right or wrong answers to the questions, because the purpose was to explore attitudes and not to promote any particular concept. All oncologists were informed and assured of their right to refuse or withdraw from the study at anytime. A closed-ended questionnaire was given to the oncologists in charge under the guidance of the research assistants to evaluate each patient (Figure 2).

### Data collection

Data considered to be associated with decision making by all the oncologists involved in this study were collected as follows. Medical insurance types were classified into 2 categories: local (medical reimbursement covered more than half of the medical expense) and nonlocal (medical reimbursement covered less than half of the medical expense). Education levels were categorized as follows: ordinary (received at least high school education) and functionally illiterate (received primary education or less). Family status was ranked as superordinate (major decision maker in the family) and subordinate (not the major decision maker in the family). The main living environment of patients was defined as the place where the patients had grown up and worked for most of their lives; this was dichotomized into city and countryside. Decision-making modes were classified into 2 conditions: patient superiority (oncologists discussed planned treatments with patients, and patients made decision themselves) and family superiority (oncologists discussed planned treatments with family members, and patients were not involved in final decision making). The above information about the patients was determined by their doctors in charge.

### Endpoints

The endpoint of this study was the patients’ ignorance of premature ending of anti-cancer therapy. Premature ending of cancer therapy means that, while the oncologist suggested continuing anti-cancer therapy, including chemotherapy, targeted therapy, radio therapy, and surgery, the anti-cancer therapy were rejected and stopped by the patients or family. Premature ending of anti-cancer therapy was considered the major moral risk in the decision making for the patients’ ADs, which might put their survival at risk [18]. This early termination of anti-cancer therapy might occur for the following reasons: 1. The family would not like to pay for the medical treatment even when they have no financial support, 2. the family would not like to take care of the patient even if they have the resources, or 3. the family wishes the patient die early to get the inheritance. Further, the premature ending of anti-cancer therapy might be against the will of the patient. However, he/she may have been told by the family that there was no need to continue anti-cancer therapy, which was not the truth. Additionally, it could be that the oncologist was not allowed to provide any information to the patient.

### Statistical analyses

We compared the variables in different groups using chi-square analysis. The criterion for statistical significance was set at α = .05, and all *P*-values were based on 2-sided tests. SPSS (version 20.0; SPSS Inc, Chicago, IL) was used for all statistical analyses.

## CONCLUSIONS

In summary, this study illustrated that ADs were not widely accepted among Chinese people. Most Chinese cancer patients were excluded from the discussion of an AD. Premature ending of anti-cancer therapy induced acceptance of ADs. Death education might improve cancer patients’ participation in AD discussions and finally help with fulfilling their personal will.
